# Facial Expression Recognition and ReHo Analysis in Major Depressive Disorder

**DOI:** 10.3389/fpsyg.2021.688376

**Published:** 2021-09-22

**Authors:** Sijia Liu, Ruihua Ma, Yang Luo, Panqi Liu, Ke Zhao, Hua Guo, Jing Shi, Fude Yang, Yunlong Tan, Shuping Tan, Zhiren Wang

**Affiliations:** ^1^Peking University HuiLongGuan Clinical Medical School, Beijing Huilongguan Hospital, Beijing, China; ^2^State Key Laboratory of Brain and Cognitive Science, Institute of Psychology, Chinese Academy of Sciences, Beijing, China; ^3^Zhumadian Psychiatric Hospital, Zhumadian, China

**Keywords:** MDD, facial expression recognition, fMRI, ReHo, cognitive

## Abstract

**Objective:** To explore the characteristics of expression recognition and spontaneous activity of the resting state brain in major depressive disorder (MDD) patients to find the neural basis of expression recognition and emotional processing.

**Methods:** In this study, two of the six facial expressions (happiness, sadness, anger, fear, aversion, and surprise) were presented in quick succession using a short expression recognition test. The differences in facial expression recognition between MDD patients and healthy people were compared. Further, the differences in ReHo values between the two groups were compared using a resting-state functional magnetic resonance imaging (fMRI) scan to investigate the characteristics of spontaneous brain activity in the resting state and its relationship with clinical symptoms and the accuracy of facial expression recognition in patients with MDD.

**Results:** (1) The accuracy of facial expression recognition in patients with MDD was lower than that of the HC group. There were differences in facial expression recognition between the two groups in sadness-anger (*p* = 0.026), surprise-aversion (*p* = 0.038), surprise-happiness (*p* = 0.014), surprise-sadness (*p* = 0.019), fear-happiness (*p* = 0.027), and fear-anger (*p* = 0.009). The reaction time for facial expression recognition in the patient group was significantly longer than that of the HC group. (2) Compared with the HC group, the ReHo values decreased in the left parahippocampal gyrus, left thalamus, right putamen, left putamen, and right angular gyrus, and increased in the left superior frontal gyrus, left middle temporal gyrus, left medial superior frontal gyrus, and right medial superior frontal gyrus in the patient group. (3) Spearman correlation analysis showed no statistical correlation between ReHo and HAMD-17 scores in MDD patients (*p* > 0.05). The ReHo value of the left putamen was negatively correlated with the recognition of fear-surprise (*r* = −0.429, *p* = 0.016), the ReHo value of the right angular gyrus was positively correlated with the recognition of sadness-anger (*r* = 0.367, *p* = 0.042), and the ReHo value of the right medial superior frontal gyrus was negatively correlated with the recognition of fear-anger (*r* = −0.377, *p* = 0.037).

**Conclusion:** In view of the different performance of patients with MDD in facial expression tasks, facial expression recognition may have some suggestive effect on the diagnosis of depression and has clinical guiding significance. Many brain regions, including the frontal lobe, temporal lobe, striatum, hippocampus, and thalamus, in patients with MDD show extensive ReHo abnormalities in the resting state. These brain regions with abnormal spontaneous neural activity are important components of LCSPT and LTC circuits, and their dysfunctional functions cause disorder of emotion regulation. The changes in spontaneous activity in the left putamen, right angular gyrus, and right medial superior frontal gyrus may represent the abnormal pattern of spontaneous brain activity in the neural circuits related to emotion perception and may be the neural basis of facial expression recognition.

## Introduction

Major depressive disorder (MDD) is a mood disorder characterized by significant and persistent mood depression accompanied by significant difficulty in emotional regulation. The latest research results of the China Mental Health Survey (Huang et al., 2019) showed that the weighted lifetime and 12-month prevalence of depression were 3.4 and 2.1%, respectively. In addition to emotional symptoms, MDD patients also suffer from cognitive decline to varying degrees, resulting in varying degrees of impairment of social function. In this situation, not only do the patients themselves struggle, but considerable burden is placed on their families and society as well.

Patients with MDD often have pessimistic thoughts, tend to interpret negative information, and have negative cognitive patterns (Joormann and Gotlib, 2007). Studies have suggested that patients with MDD exhibit excessive processing of negative emotional stimuli and increased self-attention, both of which promote negative self-related information and prolonged negative emotions (Goeleven et al., 2006; Fossati, 2018). Facial expressions, as one of the most obvious ways to identify a person’s emotional state, is an important information source in social communication and plays an essential role in daily communication. As part of social cognition, facial expression recognition is an important social skill that facilitates understanding in social interactions while also reflecting an individual’s social ability. Compared with healthy people, patients with MDD tend to evaluate positive and neutral facial expressions as sad or unhappy (Leppänen et al., 2004; Bourke et al., 2010). Further, the patient’s ability to recognize facial expressions is impaired, as is evident from the lower accuracy and longer response times when recognizing facial expressions (Delle-Vigne et al., 2014). The patients’ impaired facial expression recognition ability and negative cognitive processing bias may explain the impaired social function.

With the development of neuroimaging technology, functional magnetic resonance imaging (fMRI) technology has been increasingly applied to the study of the neural mechanisms of various brain functions and neuropsychiatric diseases. The processing of emotional information in patients with MDD has also become a research hotspot. Studies on the neuroanatomical structure of depression have found that there are brain changes related to early onset depression in the hippocampus, amygdala, caudate nucleus, putamen, and frontal cortex (Sheline, 2000). These structures are anatomically and functionally related to each other, thus constituting the limbic-cortical-striatal-pallidal-thalamic (LCSPT) circuit and the limbic-thalamic-cortical (LTC) circuit (Sheline, 2003). These are considered to be the two main neural circuits leading to the onset of depression; any brain damage involving key structures in these circuits will cause disorders of mood regulation (Price and Drevets, 2012). Current fMRI studies have shown that negative cognitive patterns in patients with MDD can manifest as abnormal connections between cognitive networks and limbic networks involved in cognitive control and self-reference processing (Fossati, 2018).

Researchers are yet to reach a unified conclusion regarding the neural mechanisms of face recognition. Some studies have found that the brain regions for facial expression recognition are widely distributed in the frontal, parietal, and anterior parts of the occipitotemporal cortex (Clark et al., 1997, 1998). Neurobiological studies on emotional perception show that the process of emotional perception may depend on the functioning of two neural systems: the ventral and dorsal. The ventral system is composed of the amygdala, insula, ventral striatum, anterior cingulate gyrus, and prefrontal cortex, which mainly recognize emotional stimuli and produce and automatically regulate emotional responses. The dorsal system, which includes the hippocampus and dorsal areas of the anterior cingulate gyrus and prefrontal cortex, is responsible for the adjustment of emotional states (Phillips et al., 2003). One hypothesis is that there are two pathways for facial expression recognition, that is, behavior-related features in the visual environment can be unconsciously detected and processed through the colliculo-pulvinar-amygdala pathway or the extrageniculostriate (collicular-thalamic-amygdala) neural pathway can process fear-related stimuli independently of the striatal cortex and normal phenomenal visual awareness (Morris et al., 2001). The conscious visual perception of the same stimulus appears to involve specific cortical regions, such as fusiform gyrus and temporal poles (Morris et al., 1999). It is suggested that the functional activation of the visual processing region may be related to the processing requirements of different expressions (Frühholz et al., 2009). The content-related activation of the visual striatum in the extracorporeal cortex may be mediated by a “top-down” mechanism in the parietal and frontal cortex that mediates long-term memory retrieval of face and object representations and maintenance through visual imagery (Ishai et al., 2000).

In order to explore the characteristics of facial expression recognition and the neural basis of emotion processing in MDD, we performed a short-term facial expression recognition test and an fMRI scan on 45 patients with depression and 24 healthy controls (HC), using regional homogeneity (ReHo) to (1) compare the differences in facial expression recognition between patients with MDD and healthy people and (2) to explore the characteristics of spontaneous brain activity in the resting state of patients with MDD and their relationship with clinical symptoms and facial expression recognition accuracy.

## Materials and Methods

### Participants

A total of 45 patients with MDD and 24 HC participants were recruited for this study. Patients with MDD were recruited from the Zhumadian Psychiatric Hospital, and healthy subjects were recruited from surrounding communities.

Inclusion criteria for patients with MDD:

(1) The DSM-IV (Diagnostic and Statistical Manual, Fourth Edition) criteria for depression and does not incorporate other Axis I or II diagnoses; (2) have not used antidepressants for at least 2 months before the scan; (3) HAMD17 ≥ 17 points; (4) right-handed; (5) Han nationality; (6) 15–50 years old.

Exclusion criteria for patients with MDD:

(1) Complicated with other mental disorders; (2) history of organic brain disease, craniocerebral injury, electroconvulsive therapy, or other serious physical diseases; (3) history of alcohol and substance abuse; (4) intellectual disability; (5) pregnant and lactating women; (6) claustrophobia; (7) any contraindication to magnetic resonance.

Inclusion criteria for HC:

(1) Have not suffered from any previous mental disorders; (2) HAMD17 < 7 points; and (3) negative family history of mental disorders.

Exclusion criteria for HC:

(1) First-degree relatives have been diagnosed with mental illness; (2) history of organic brain disease, craniocerebral injury, electroconvulsive therapy, or other serious physical diseases; (3) history of alcohol and substance abuse; (4) intellectual disability; (5) pregnant and lactating women; (6) claustrophobia; and (7) any contraindication to magnetic resonance.

This study was reviewed and approved by the Ethics Committee of Beijing Huilongguan Hospital and the Ethics Committee of Zhumadian Psychiatric Hospital (Ethical approval number: 2016-72). Prior to the study, all the subjects and their legal guardians were informed in detail about the content and possible risks and benefits of participating in the study. Participation was voluntary and all participants signed an informed consent form.

### Research Tools

1. Structured Clinical Interview for DSM-IV-TR Axis I Disorders-Patient Edition (SCID-I/P): Used for diagnostic evaluation of subjects; Structured Clinical Interview for DSM-IV-TR Axis I Disorders—Non-Patient Edition (SCID-I/NP): Used for the screening of healthy controls (Lobbestael et al., 2011).
2. Edinburgh Handness Inventory (EHI): Used to evaluate whether a subject is left- or right-handed (Oldfield, 1971).
3. Hamilton Depression Rating Scale for Depression-17 (HAMD-17): Used to assess the severity of a subject’s depressive disorder, it consists of 17 items (Hamilton, 1960).
4. Hamilton Anxiety Scale (HAMA): Includes 14 items and is used to assess the severity of subjects’ anxiety (Thompson, 2015).
5. Young Mania Rating Scale (YMRS): Used to assess the severity of the subjects’ manic symptoms (Young et al., 1978).
6. Family History Research Diagnostic Criteria (FHRDC): Used to exclude members of the HC group with a family history of mental illness among the first-degree relatives (Andreasen et al., 1977).
7. MRI Safety Questionnaire:Used to evaluate whether subjects are suitable for MRI examination to ensure the personal safety of the subject.

### Facial Expression Recognition Experimental Stimulus Paradigm

Ten models were selected from the Ekman gallery (Ekman and Friesen, 1976), with each model comprising six types of emotions indicated by facial expressions (happiness, sadness, anger, fear, aversion, and surprise) for a total of 60 pictures. The experimental paradigm was compiled and run with E-prime 2.0 (psychology software tools). The experimental instrument used was a Dell laptop. The display is a 16-inch (31 cm × 17.5 cm) built-in display with a refresh rate of 60 Hz and a resolution of 1,280 pixels × 800 pixels. The subjects’ eyes were placed approximately 60 cm away from the center of the computer screen. In each block, there were 20 pictures of two types of facial expressions. After the participant pressed the “space” key, a fixation point “+” was presented to the subject at the center of the screen for 200 ms. After the fixation point disappeared, an emotional expression picture of a model was presented, and the presentation time was randomly configured to be either 100 or 300 ms. Both the expression picture and presentation time appeared randomly. Participants were asked to select either one or both of the two given expression options to judge the expression presented. Participants needed to make a judgment within 3 s after the picture was presented; otherwise, it would automatically skip to the next picture, and the judged face would be counted as an error.

**FIGURE 1:**
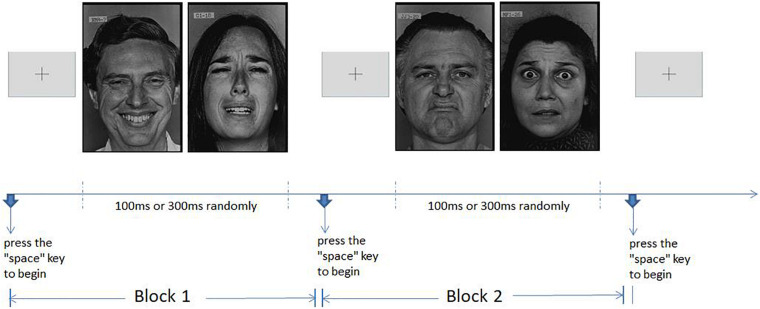
Graph of experiment stimulation program.

In each set of tests, for example, to identify happy and sad expressions, two expressions of 10 models appear randomly 20 times in total. Among the options, 1 represents happy and 2 represents sad, and participants can make a judgment choice. After a set of tasks is completed, participants press the “space” key to start the next set. As shown in Figure 1, the six expressions are combined in pairs, there are a total of 15 groups, and each group contains two expressions and a total of 20 pictures.

### fMRI Data Collection and Processing

All subjects were examined using a GE HCGEHC 3.0T MRI scanner (Signa HDXT, GE, United States) at Zhumadian Psychiatry Hospital. During the scan, subjects were required to lie on their backs, close their eyes, keep their head fixed, stay awake without performing specific cognitive recognition, and use foam pads and headsets provided by the manufacturer to limit head movement and thus reduce noise. After completing the brain anatomy and positioning, a resting state fMRI scan was performed using the echoplanar imaging sequence. The scanning scope was the whole brain, and the scanning content was a high-resolution T1 structural image and resting state functional image. The relevant scan parameters were as follows: TR = 2,000 ms, TE = 30 ms, FA = 90°, FOV = 22 cm, 64 × 64 matrix, 33 slices, slice thickness = 4 mm, and 210 volumes.

The original images of the subjects were converted into NIFTI format through the dcm2niigui function of the Micron software, and the fMRI data processing assistant DPARSFA3.2 (data processing assistant for resting state fMRI advanced edition) in the DPABI1.3 software^1^ and SPM12 (statistical parametric mapping,^2^) were run on the Maltab R2017a platform R2017a^3^ to preprocess the data after converting format. The specific process includes the following steps:

(1) Discard the first ten volumes to exclude the influence of machine signal instability and the subject’s adaptation process on the results.

(2) Slice timing: Correct the difference in time between the acquired images of each slice.

(3) Realign: Exclude subjects with head movement >1.5 mm and rotation >1.5° to reduce the influence of head movement noise on the signal.

(4) Normalization: Normalize images to the standard echoplanar imaging template, and resample each voxel to 3 × 3 × 3 mm^3^.

(5) Coregister: Coregister the functional image and anatomical image (T1-weighted image) to accurately locate the functional activation area.

(6) Detrend and filter (0.01–0.08 Hz): Reduce the influence of low-frequency linear drift and high-frequency physiological noise (e.g., breathing, heartbeat, etc.).

The ReHo brain map was constructed by calculating the Kendall coefficient of the time series consistency between each voxel and its neighboring voxels. The ReHo value of each voxel was subtracted from the average ReHo value of the whole brain and then divided by the standard deviation for subsequent analysis. The KCC-ReHo value of all single voxel directions was calculated and normalized to the KCC-ReHo z value (Zuo et al., 2013). A Gaussian kernel with a full width and half height of 4 mm was used for smoothing to reduce the influence of deformation and noise in the process of standardization, improve the signal-to-noise ratio and statistical efficiency, and enhance the image effect.

Using DPABI1.3 software, a two-sample *t*-test was performed on the ReHo graphs of the patient group and the HC group. Gender, age, and head movement of the subjects were controlled as covariates. Brain templates were selected to overlay, and Alphasim correction was performed. The correction threshold was *P* < 0.01, Monte Carlo simulation was performed 1,000 times, the brain regions with differences between the groups were extracted as the template mask, the ReHo value of each subject was extracted according to the template, and Spearman correlation analysis was performed on the ReHo value and HAMD-17 score of the patient group. *P* < 0.05 was considered statistically significant.

### Statistics

#### Demographic and Clinical Data

SPSS 20.0 was used for statistical analysis, counting data was expressed by cases, and the chi-square test was used for comparison between groups. Data with a normal distribution were expressed by $\bar{x}$ ± s deviation, data that did not conform with normal distribution were expressed by the median (lower quartile, upper quartile). *P* < 0.05 was regarded as statistically significant.

#### Facial Expression Recognition Data Analysis

According to the signal detection theory, we used d′ to express the accuracy of facial expression recognition, namely the measured value of discrimination ability, and used the hit rate and false alarm rate to estimate the recognition ability. The d′ values of the two groups of facial expressions were analyzed using a non-parametric test. To assess the difference in facial expression recognition speed between depressed patients and healthy controls, non-parametric tests were used to analyze the response time (RT) for facial expression recognition between the two groups.

The formula for calculating the accuracy of facial expression recognition is as follows: d′ = zH-zFA, where d′ represents accuracy, z represents the standard deviation, zH is the hit rate, and zFA is the false alarm rate.

## Results

### Comparison of General Demographic and Clinical Data

There were 45 and 24 subjects in the depression and healthy control group, respectively. Among them, one patient with MDD showed demyelination of white matter on MRI, two patients with MDD did not adhere to the MRI examination, and one MDD patient received MECT treatment. One HC was excluded due to being an ethnic minority. Several subjects with head movement (>1.5 mm and rotation > 1.5°) were also excluded, including seven patients in the MDD group and four subjects in the HC group. Finally, the subjects of the statistical analysis were 35 patients in the depression group and 19 subjects in the control group. There were no statistically significant differences in sex, age, and years of education between the two groups (*P* > 0.05). The general information and clinical evaluation scales of the two groups are shown in Table 1.

**TABLE 1:** Comparison of general demographic data and clinical symptom scores (x ± s).

	MDD (*n* = 35)	HC (*n* = 19)	χ2/t/F	p
Sex (male/female)*	22/13	7/12	3.35	0.07
Age (year)	26.17 ± 9.80	28.16 ± 8.05	2.26	0.45
Education (year)	11.83 ± 2.78	13.11 ± 3.03	1.17	0.13
Duration (month)	24(8, 48)			
HAMD-17 (score)	20(18, 23)			
HAMA (score)	21.88 ± 8.38			
YMRS (score)	2.40 ± 1.70			

* is the chi-square test, the rest is the independent sample *t* test. HAMD-17, Hamilton Depression Scale; HAMA, Hamilton Anxiety Scale; YMRS, Young’s Mania Scale.

### Facial Expressions

**FIGURE 2:**
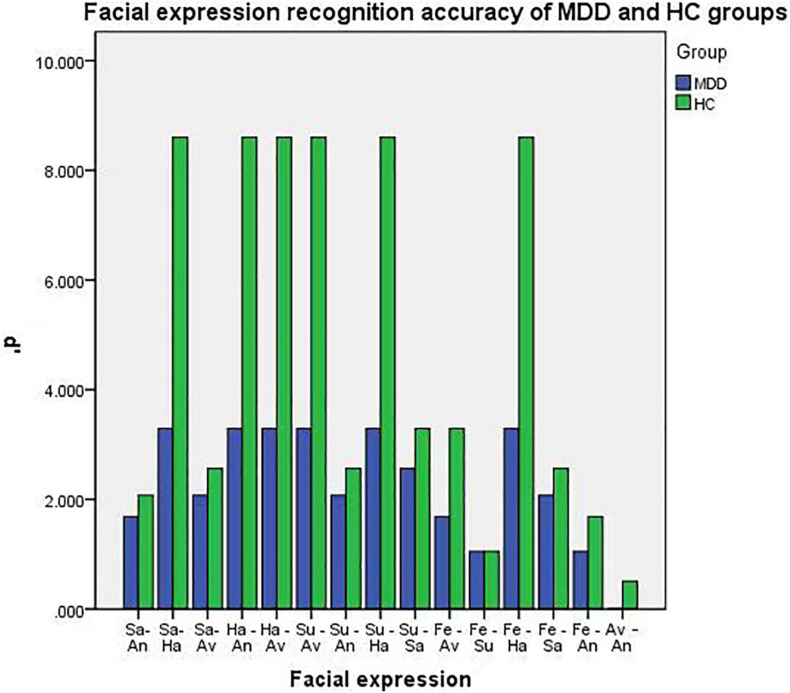
Facial expression recognition accuracy of MDD and HC groups.

Differences were found in facial expression recognition between the two groups in sadness-anger (*P* = 0.026), surprise-aversion (*P* = 0.038), surprise-happiness (*P* = 0.014), surprise-sadness (*P* = 0.019), fear-happiness (*P* = 0.027), and fear-anger (*P* < 0.009). The accuracy of facial expression recognition in patients with MDD was lower than that of the HC group, as shown in Table 2 and Figure 2.

**TABLE 2:** Facial expression recognition d′ values of MDD and HC groups.

	MDD (*n* = 35)	HC (*n* = 19)	*P*
Sa-An	1.68 (0.77, 2.07)	2.07 (1.05, 2.56)	0.026*
Sa-Ha	3.29 (2.56, 8.60)	8.60 (3.29, 8.60)	0.325
Sa-Av	2.07 (1.35, 3.29)	2.56 (2.07, 3.29)	0.144
Ha-An	3.29 (2.56, 8.60)	8.60 (3.29, 8.60)	0.080
Ha-Av	3.29 (2.56, 8.60)	8.60 (3.29, 8.60)	0.098
Su-Av	3.29 (2.07, 8.60)	8.60 (3.29, 8.60)	0.038*
Su-An	2.07 (1.35, 2.56)	2.56 (1.68, 3.29)	0.153
Su-Ha	3.29 (2.56, 8.60)	8.60 (3.29, 8.60)	0.014*
Su-Sa	2.56 (2.07, 3.29)	3.29 (2.56, 8.60)	0.019*
Fe-Av	1.68 (1.05, 3.29)	3.29 (1.68, 8.60)	0.066
Fe-Su	1.05 (0.51, 1.68)	1.05 (0.77, 1.68)	0.250
Fe-Ha	3.29 (2.56, 8.60)	8.60 (3.29, 8.60)	0.027*
Fe-Sa	2.07 (1.35, 3.29)	2.56 (1.68, 3.29)	0.226
Fe-An	1.05 (0.51, 2.56)	1.68 (1.35, 2.56)	0.009*
Av-An	0 (−0.51, 0.51)	0.51 (−0.25, 1.35)	0.186

*p < 0.05.

**FIGURE 3:**
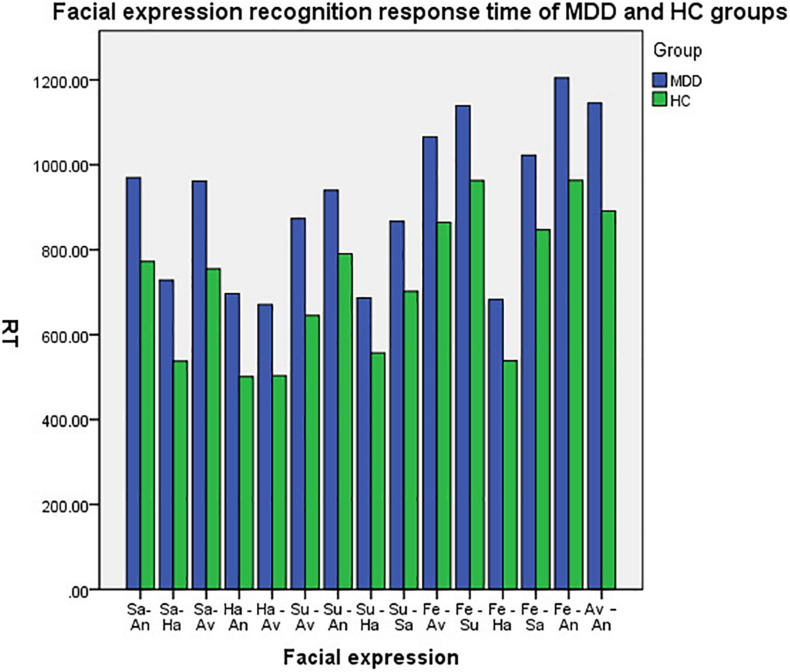
Facial expression recognition response time of MDD and HC groups.

There are significant differences in the reaction time of facial expression recognition between the two groups (*P* < 0.05); the reaction time of facial expression recognition in the patients with MDD group is longer than that of the HC group, and the reaction time of the two groups in identifying facial expressions (sadness-happiness, happiness-anger, happiness-aversion, surprise-happiness, fear-happiness) are shorter than that of other expressions, as shown in Table 3 and Figure 3 below.

**TABLE 3:** Facial expression recognition response time of MDD and HC groups.

	MDD (*n* = 35)	HC (*n* = 19)	*p*
Sa-An	970.00 (637.50, 1,436.00)	772.50 (566.00, 1,097.50)	0.000
Sa-Ha	728.50 (514.00, 1,052.50)	537.50 (420.00, 682.00)	0.000
Sa-Av	962.00 (664.75, 1,383.50)	755.00 (555.25, 1,131.00)	0.000
Ha-An	696.50 (502.25, 983.50)	501.00 (381.75, 700.00)	0.000
Ha-Av	671.00 (485.25, 963.25)	503.00 (383.00, 662.50)	0.000
Su-Av	873.50 (641.25, 1,261.75)	645.00 (478.50, 919.50)	0.000
Su-An	940.00 (656.00, 1,365.00)	790.50 (549.50, 1,146.25)	0.000
Su-Ha	686.50 (486.25, 987.75)	557.00 (412.25, 719.50)	0.000
Su-Sa	867.00 (609.25, 1,305.50)	702.00 (504.75, 992.00)	0.000
Fe-Av	1,066.00 (758.50, 1,525.25)	864.00 (610.25, 1,236.00)	0.000
Fe-Su	1,139.00 (825.25, 1,707.75)	963.00 (656.50, 1,390.75)	0.000
Fe-Ha	682.50 (499.25, 969.00)	538.50 (419.00, 700.75)	0.000
Fe-Sa	1,022.50 (671.50, 1,453.50)	847.00 (564.25, 1,256.00)	0.000
Fe-An	1,205.00 (830.25, 1,692.00)	963.50 (681.50, 1,421.50)	0.000
Av-An	1,145.50 (785.75, 1,685.50)	891.00 (637.00, 1,347.25)	0.000

### Brain Area With Abnormal ReHo Value in Major Depressive Disorder Patients

**FIGURE 4:**
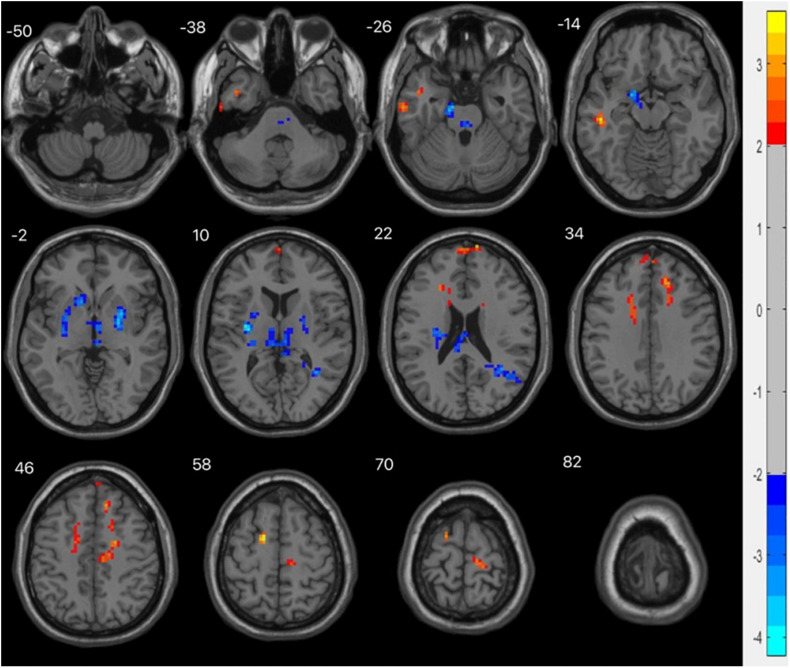
fMRI shows brain regions with abnormal ReHo values in the MDD group compared with the HC group. The chromatographic value in the figure is the t value, indicating the level of ReHo. The red is the area where the ReHo value is significantly increased, and the blue is the area where the ReHo value is significantly decreased.

Alphasim correction was used to analyze the ReHo values (*P* < 0.01, cluster size > 123). Compared with the HC group, the ReHo values decreased in the left parahippocampal gyrus, left thalamus, right putamen, left putamen, and right angular gyrus, and increased in the left superior frontal gyrus, left middle temporal gyrus, left medial superior frontal gyrus, and right medial superior frontal gyrus, as shown in Table 4 and Figure 4.

**TABLE 4:** Brain area with abnormal ReHo value in MDD patients.

Brain area	BA	Cluster size	MNI			*t*
			X	Y	Z	
ParaHippocampal_L	34	150	−15	−15	−24	–3.862
Temporal_Mid_L	20	136	−60	−12	−30	3.4777
Thalamus_L	23	267	−6	−27	18	–3.5041
Putamen_R	–	125	24	−6	0	–3.846
Putamen_L	13	214	−33	−9	9	–4.2369
Frontal_Sup_Medial_L	9	130	−3	57	42	3.4201
Angular_R	39	132	36	−51	18	–3.5399
Frontal_Sup_L	6	184	−15	0	57	3.6504
Frontal_Sup_Medial_R	6	277	9	−21	51	3.3734

### Correlation Between ReHo Value and Clinical Symptoms

Spearman correlation analysis between ReHo and HAMD-17 scores in brain areas with significant differences in MDD patients showed that there was no statistical correlation between ReHo and HAMD-17 scores in MDD patients (*p* > 0.05), as shown in Table 5.

**TABLE 5:** Correlation between ReHo value and clinical symptoms.

Brain area	*r*	*p*
ParaHippocampal_L	–0.081	0.665
Temporal_Mid_L	–0.170	0.360
Thalamus_L	0.006	0.976
Putamen_R	0.161	0.386
Putamen_L	0.264	0.151
Frontal_Sup_Medial_L	–0.344	0.058
Angular_R	0.238	0.197
Frontal_Sup_L	–0.150	0.422
Frontal_Sup_Medial_R	–0.055	0.770

**p* < 0.05.*

### Correlation Between ReHo Value and Facial Expression Recognition Accuracy

Spearman correlation analysis between the ReHo value of the brain regions with significant differences and the recognition accuracy of facial expressions in MDD patients showed that the ReHo value of the left putamen was negatively correlated with the recognition of fear-surprise (*r* = −0.429, *p* = 0.016), while that of the right angular gyrus was positively correlated with the recognition of sadness-anger (*r* = 0.367, *p* = 0.042), and the ReHo value of the right medial superior frontal gyrus was negatively correlated with the recognition of fear-anger (*r* = −0.377, *p* = 0.037), as shown in Table 6.

**TABLE 6:** Correlation between ReHo value and facial expression recognition accuracy.

	ROI1		ROI2		ROI3		ROI4		ROI5	
	*r*	*p*	*r*	*p*	*r*	*p*	*r*	*p*	*r*	*p*
Sa-An	0.346	0.056	–0.293	0.109	0.067	0.721	0.150	0.422	0.100	0.592
Sa-Ha	0.004	0.985	–0.091	0.627	0.143	0.443	0.025	0.893	0.108	0.565
Sa-Av	0.144	0.439	–0.129	0.488	0.174	0.350	0.354	0.050	–0.037	0.842
Ha-An	0.071	0.705	0.112	0.549	0.148	0.428	0.261	0.156	–0.056	0.763
Ha-Av	0.003	0.988	–0.019	0.921	0.056	0.764	0.155	0.406	0.150	0.421
Su-Av	0.181	0.329	–0.034	0.856	0.191	0.304	0.171	0.359	–0.036	0.846
Su-An	0.125	0.502	–0.171	0.359	0.295	0.107	0.279	0.129	0.071	0.705
Su-Ha	–0.066	0.723	–0.018	0.925	0.232	0.209	0.234	0.205	–0.124	0.505
Su-Sa	0.321	0.078	–0.251	0.173	–0.023	0.903	0.248	0.179	–0.137	0.463
Fe-Av	0.133	0.476	–0.067	0.722	0.052	0.781	0.277	0.131	–0.171	0.357
Fe-Su	–0.028	0.882	0.166	0.371	–0.175	0.345	–0.091	0.627	−0.429*	0.016
Fe-Ha	0.017	0.927	–0.100	0.592	–0.168	0.367	0.009	0.960	–0.032	0.865
Fe-Sa	0.100	0.594	–0.216	0.243	0.271	0.141	0.260	0.157	0.043	0.820
Fe-An	0.279	0.129	–0.161	0.386	0.117	0.530	0.172	0.354	–0.017	0.929
Av-An	–0.025	0.892	–0.274	0.136	0.032	0.865	0.043	0.818	–0.038	0.839

	**ROI6**		**ROI7**		**ROI8**		**ROI9**			
	***r***	***p***	***r***	***p***	***r***	***p***	***r***	***p***		

Sa-An	–0.198	0.287	0.367*	0.042	0.133	0.476	–0.062	0.741		
Sa-Ha	0.087	0.643	–0.035	0.853	0.056	0.764	0.054	0.774		
Sa-Av	–0.179	0.336	–0.098	0.600	–0.103	0.582	–0.167	0.369		
Ha-An	0.075	0.688	0.027	0.885	–0.006	0.972	0.130	0.487		
Ha-Av	0.209	0.260	0.097	0.603	0.056	0.764	0.081	0.667		
Su-Av	–0.002	0.989	0.000	0.999	–0.043	0.818	–0.171	0.359		
Su-An	–0.020	0.917	–0.245	0.185	–0.102	0.587	0.185	0.320		
Su-Ha	0.215	0.246	–0.060	0.750	0.317	0.082	0.150	0.422		
Su-Sa	–0.258	0.161	0.009	0.962	–0.117	0.530	–0.106	0.570		
Fe-Av	–0.128	0.494	–0.018	0.924	–0.055	0.770	–0.084	0.655		
Fe-Su	–0.209	0.259	0.073	0.696	0.127	0.496	–0.084	0.654		
Fe-Ha	–0.113	0.544	0.220	0.235	0.013	0.943	0.033	0.861		
Fe-Sa	–0.106	0.569	–0.030	0.871	–0.133	0.474	–0.163	0.380		
Fe-An	–0.136	0.465	–0.116	0.533	–0.258	0.161	−0.377*	0.037		
Av-An	–0.154	0.408	–0.181	0.330	0.041	0.828	0.162	0.383		

*P < 0.05.

*ROI1, ParaHippocampal_L; ROI2, Temporal_Mid_L; ROI3, Thalamus_L; ROI4, Putamen_R; ROI5, Putamen_L; ROI6, Frontal_Sup_Medial_L; ROI7, Angular_R; ROI8, Frontal_Sup_L; ROI9, Frontal_Sup_Medial_R.*

## Discussion

In this study, six expressions (happiness, sadness, anger, fear, aversion, and surprise) from the Ekman library were selected, and the pairwise comparison paradigm was used to perform facial expression recognition experiments on MDD patients and healthy controls. The accuracy of facial expression recognition and reaction time was used to explore the characteristics of facial expression recognition in patients with depression. It was found that the accuracy of facial expression recognition in MDD patients was generally lower than that of the healthy control group, and the reaction time of facial expression recognition was longer than that of the healthy control group. There were significant differences in facial expression recognition between the two groups in sadness and anger, surprise and aversion, surprise and happiness, surprise and sadness, fear and happiness, and fear and anger. Patients with MDD tend to identify positive expressions as neutral expressions and neutral expressions as negative expressions, indicating that the impaired facial recognition ability of patients with MDD is mainly manifested in the recognition of negative expressions. The MDD patients’ recognition of surprise-aversion, surprise-happiness and surprise-sadness was significantly reduced, and the patients were more likely to mistake surprise for other expressions, suggesting that surprise may be a more complex expression that is more difficult for them to recognize.

This study found that the reaction time of facial expression recognition in patients with MDD was longer than that of the healthy control group. Among them, the reaction times of the two groups in identifying facial expressions (sadness-happiness, happiness-anger, happiness-aversion, surprise-happiness, fear-happiness) was shorter than that of other expressions. Previous studies on the development of emotional expression recognition have shown that the ability to recognize emotional expressions improves with age; children first recognize positive expressions with the highest accuracy followed by negative expressions, and finally neutral expressions, which are more difficult to perceive (Herba and Phillips, 2004). Adults recognize facial expressions more accurately and faster than children, and the speed of information processing varies with emotion (fastest for happiness, slowest for fear), particularly for negative facial expressions (De Sonneville et al., 2002). In general, slower emotion recognition may seriously impede the social communication and development of patients. The general prolongation of the reaction time of facial expression recognition indicates that there is a widespread reaction delay in patients with MDD, which is also consistent with the clinical symptoms of retardation of thinking and affective flattening.

Similar to our research results, Gollan et al. (2008) explored the differences in emotional information processing between patients with MDD and healthy controls. Patients with MDD mainly showed defects in facial expression recognition, intensity classification, recognition of emotional significance information, and reaction time for neutral information. Depression has significant effects on the perceived intensity of negative emotional stimuli, delayed speed in processing sad emotional information, and the interpretation of neutral faces as sadness. Tong et al. (2020) discussed the bias in the classification and processing of happy faces in patients with MDD and found that the total response time of classified faces in the depression group was longer than that of the control group and the accuracy rate was lower than that of the control group. When classifying happy faces, the amplitude of N170 in the depression group decreased and the latency of some brain regions was prolonged, suggesting that the cognitive bias of MDD patients may be related to long-term positive facial information processing and the difficulty in generating positive emotional responses. The results of a meta-analysis on the degree of attentional bias to negative stimuli in patients with MDD supported the existence of attentional bias to negative information, and the conclusions are independent of age, sex, type of depression sample, year of publication, time of stimulus presentation, or influence of stimulus type (Peckham et al., 2010). These studies suggest that patients with MDD have a higher sensitivity to negative emotions and experience difficulty in processing positive emotions, leading to significant and persistent depression and difficulty in regulating emotions, which may be the pathophysiological basis of the disease.

At the neurological level, the excessive attention of depressed individuals to negative information is believed to stem from excitement and inhibitory dysfunction (Zhang et al., 2019). Some scholars have used the return inhibition paradigm to confirm that patients with MDD show an obvious lack of attention inhibition to negative emotional faces, and insufficient inhibition of negative stimuli cannot eliminate the interference of negative stimuli, leading to the maintenance and development of depression (Dai and Feng, 2009). The hypersensitivity of patients with depression to negative events may disappear in the remission period or be further enhanced when depression recurs (Nandrino et al., 2004). Inadequate suppression of negative emotional information has also been observed in individuals with depressive tendencies who have not been diagnosed with depression (Zhang et al., 2019). The above studies suggest that facial expression recognition can be used as an effective tool to distinguish patients with depression and people with depressive tendencies from the general population. An awareness of the characteristics of facial expression recognition in patients with depression could provide a basis for early intervention in cases of depression.

ReHo is the consistency and synchronization between a voxel and its surrounding voxels in a time series, which is an indicator of the activity of local brain regions. The higher the ReHo value, the more consistent the time series of local neuron activity, while a decrease in ReHo indicates that the local neuron activity tends to be disordered over time. Abnormal ReHo values can reflect abnormal generation and regulation mechanisms of neuronal synchronous activity in this brain region. We find that compared with healthy controls, the abnormal ReHo values of the whole brain in MDD patients are widely distributed in multiple brain regions in the resting state. There are dual phenomena of increased and decreased ReHo values in the left parahippocampal gyrus, left thalamus, putamen, and right angular gyrus, while the ReHo values of the left superior frontal gyrus, left middle temporal gyrus, and medial superior frontal gyrus all increased. These brain regions with abnormal ReHo values are an important part of the LCSPT and LTC circuits, which is consistent with previous studies.

The putamen is part of the striatum, and abnormalities in the striatum play a role in mood and cognitive changes associated with depression (Furuyashiki and Deguchi, 2012). In this study, there was decreased consistency of spontaneous neural activity in the left putamen in the basal state in patients with MDD, and the ReHo value of the left putamen was negatively correlated with the recognition of fear-surprise (*r* = −0.429, *P* = 0.016), suggesting that the decline in the ability to process negative expression information in patients with MDD is closely related to the abnormal function of the left putamen. Further studies are needed to confirm these findings.

The angular gyrus is the visual language center located in the posterior part of the inferior parietal lobule; it emerges as a cross-modal hub that combines multiple sensory information. Lesions in the angular gyrus lead to further declines in cognitive function (Seghier, 2013). We found that the ReHo value of the right angular gyrus decreased in patients with MDD. In addition, in the analysis of the correlation between the ReHo value of patients with MDD and the accuracy of facial expression recognition, we found that the ReHo value of the right angular gyrus was positively correlated with the recognition of sadness-anger (*r* = 0.367, *P* = 0.042), suggesting that dysfunction of the angular gyrus may be the abnormal manifestations of the LCSTC and LCSPT neural circuits in patients with MDD. The decrease in spontaneous activity of the right angular gyrus may lead to a weakened ability of negative expression information processing in patients.

The frontal lobe, which is involved in emotion recognition, emotion processing, and emotion behavior generation, is an important hub of LCSPT and LTC circuits and the brain region that has received the most attention. The prefrontal lobe is an important brain region for emotion processing, being divided into the medial superior frontal gyrus, orbital superior frontal gyrus, dorsolateral superior frontal gyrus, and orbital superior frontal gyrus. The medial superior frontal gyrus is part of the default mode network and is involved in a variety of emotional processes. Structural damage and dysfunction of the medial superior frontal gyrus are related to emotional regulation disorders. In this study, it was found that the ReHo values of the left superior frontal gyrus, left medial superior frontal gyrus, and right medial superior frontal gyrus increased, suggesting that the superior frontal gyrus may have a compensatory effect on other brain regions in the emotional regulation circuit. The ReHo value of the right medial superior frontal gyrus was negatively correlated with the recognition of fear-anger (*r* = −0.377, *P* = 0.037), suggesting that dysfunction of the medial prefrontal cortex may be related to bias in the cognitive processing of negative emotions in patients with MDD.

Multiple brain regions may be involved in the recognition of abnormal facial expressions in patients with MDD. When performing facial expression recognition tasks, these brain regions are activated to varying degrees, depending on the emotion type presented and the process being evaluated (i.e., emotion recognition vs. experiencing emotion vs. regulating emotion experience) and the cognitive requirements of the task (Phillips et al., 2003). The changes in spontaneous activity in the left putamen, right angular gyrus, and right medial superior frontal gyrus were correlated with the accuracy of facial expression recognition, suggesting that these brain regions may have an impact on the potential neural connection of emotion perception.

The results of this study show that no statistical correlation between the ReHo value of patients with MDD and the severity of depressive symptoms, suggesting that this difference may be related to the disease itself, has nothing to do with its clinical symptoms, and may be an indicator of its diathology.

## Conclusion

This study combined behavioral studies of facial expression recognition cognition with brain function imaging studies and found a decreased accuracy of facial expression recognition in patients with MDD, prolonged reaction time for facial expression recognition, and impaired facial recognition ability, mainly in the recognition of negative expressions. In view of the different performance of patients with MDD in facial expression tasks, facial expression recognition may have some suggestive effect on the diagnosis of depression and has clinical guiding significance. Many brain regions, including the frontal lobe, temporal lobe, striatum, hippocampus, and thalamus, in patients with MDD show extensive ReHo abnormalities in the resting state. These brain regions with abnormal spontaneous neural activity are important components of LCSPT and LTC circuits, and their dysfunctional functions lead to disorders in emotion regulation. The reduction of spontaneous activity in the left putamen, right angular gyrus, and right medial superior frontal gyrus may represent an abnormal pattern of spontaneous brain activity in the neural circuits related to emotion regulation, which may be the neural basis of facial expression recognition.

Given the relatively small sample size and the exploratory nature of these analyses, there were no corrections for multiple comparisons. Although the significance of the reported *p*-values is potentially inflated, the data presented in the current study can be considered as being a reasonably robust representation of the relationships between the variables of interest. It is necessary for future research to further expand the sample size to verify the repeatability of the results. In addition, the relationship between neuroimaging results and clinical symptoms requires further study. Future studies should consider selecting first-episode untreated patients or high-risk groups for follow-up studies, using task-state magnetic resonance technology to scan the participants during the implicit processing of different emotional faces, and utilizing structural images, DTI, and other modal data, or electroencephalogram, event-related potential, and magneto encephalography to examine neural activities associated with facial expressions.

## Data Availability Statement

The original contributions presented in the study are included in the article/supplementary material, further inquiries can be directed to the corresponding author/s.

## Ethics Statement

The studies involving human participants were reviewed and approved by the studies involving human participants were reviewed and approved by Research Ethics Committee at the Beijing Huilongguan Hospital and Zhumadian Psychiatric Hospital. The patients/participants provided their written informed consent to participate in this study. Written informed consent to participate in this study was provided by the participants’ legal guardian/next of kin. Written informed consent was obtained from the individual(s) for the publication of any potentially identifiable images or data included in this article.

## Author Contributions

SL participated in the topic selection and design, research implementation, data collection, data analysis and interpretation, and manuscript drafting. ZW participated in the topic selection and design of the manuscript, revising the key conclusions in the manuscript, and obtaining research funds. KZ participated in the topic selection and design of the manuscript, as well as the analysis and interpretation of guiding materials. JS, PL, RM, and YL carried out research and collected data. FY, YT, ST, and HG provided administrative, technical, and material support and reviewed the content critically. All authors contributed to the article and approved the submitted version.

## Conflict of Interest

The authors declare that the research was conducted in the absence of any commercial or financial relationships that could be construed as a potential conflict of interest.

## Publisher’s Note

All claims expressed in this article are solely those of the authors and do not necessarily represent those of their affiliated organizations, or those of the publisher, the editors and the reviewers. Any product that may be evaluated in this article, or claim that may be made by its manufacturer, is not guaranteed or endorsed by the publisher.

## Footnotes

1
http://rfmri.org/dpabi

2
https://www.fil.ion.ucl.ac.uk/spm/software/spm12/

3
https://www.mathworks.com/
